# Preliminary assessment of the degree of food addiction through the Spanish Yale Food Addiction Scale for Children (S-YFAS-C) in a pilot pediatric population

**DOI:** 10.1186/s40337-023-00798-9

**Published:** 2023-05-11

**Authors:** Néstor Benítez-Brito, Himar González-Pacheco, Berta Pinto-Robayna, Francisco Moreno-Redondo, Carlos Díaz-Romero, Yolanda Ramallo-Fariña

**Affiliations:** 1 Chemistry Section, Department of Chemical Engineering and Pharmaceutical Technology, Nutrition and Bromatology Area, Pharmacy Faculty, Science Faculty, University of La Laguna, Avenida Astrofísico Francisco Sánchez, s/n, Apartado 456, San Cristóbal de La Laguna, S/C of Tenerife, 38200 Tenerife, Spain; 2 Canary Islands Health Research Institute Foundation (FIISC), Evaluation Unit (SESCS), Canary Islands Health Service (SCS), Tenerife, Spain; 3 Network for Research On Chronicity, Primary Care, and Health Promotion (RICAPPS), Tenerife, Spain

**Keywords:** Addiction, Eating disorders, Food addiction, Substance dependence, Substance-use disorders, S-YFAS-C

## Abstract

**Introduction:**

The *Spanish Yale Food Addiction Scale for Children* (S-YFAS-C) scale is the first tool adapted to Spanish to evaluate food addiction (FA) in the paediatric population. The aim of this study is to preliminarily evaluate the degree of FA in a non-clinical pilot paediatric population.

**Material and methods:**

A transversal observational study was performed on a convenience sample comprised of boys and girls aged 9 to 12 (4th to 6th year primary school). The main outcome measures were evaluation of FA (S-YFAS-C scale), child feeding attitudes (ChEAT scale) and evaluation of body image (CDRS scale). Moreover, sociodemographic and anthropometric data were collected. A descriptive and bivariate analysis of the main characteristics of subjects and outcome measures was performed.

**Results:**

A total of 21 boys and 24 girls were preliminarily evaluated and the minimum and maximum values obtained were for age (9.48–12.33), weight in kilograms (26.6–64.5), height in centimetres (131–163), BMI (14.2–27.9) and BMI Z-score (−1.36–2.66). The average number of FA symptoms measured with the S-YFAS-C scale is 1.67 ± 1.45 (range 0–7). A total of 20% of the sample shows three or more symptoms for FA, risk of developing a food disorder and distortion of the body image. Moreover, statistically significant differences were observed between desired body image in boys and girls (*P* = 0.001).

**Conclusions:**

The S-YFAS-C scale enables evaluating food addiction in Spanish-speaking boys and girls. The data obtained in regard to quantifying symptoms are similar compared to the original scale (S-YFAS-C: 1.67 ± 1.45 vs. YFAS-C: 2 ± 1.81). The option to score the counting of symptoms is the most sensitive measure to evaluate subclinical food behaviours.

## Abstract (summary)

The *Spanish Yale Food Addiction Scale for Children* (S-YFAS-C) scale is the first tool adapted to Spanish to evaluate food addiction (FA) in the paediatric population. The aim of this study is to preliminarily evaluate the degree of FA in a non-clinical pilot paediatric population. A total of 21 boys and 24 girls were preliminarily evaluated and 20% of the sample shows three or more symptoms for FA, risk of developing a food disorder and distortion of the body image. The S-YFAS-C scale enables evaluating food addiction in Spanish-speaking boys and girls. The data obtained in regard to quantifying symptoms are similar compared to the original scale.

## Introduction

In the last few years there has been emerging research that reflects similarities between addiction to psychoactive substances and excessive food consumption [1]. This concept, called food addiction (FA), has led to the scientific community starting to perform new studies around this hypothesis, which makes reference to that food behaviour that entails an apparently uncontrollable excessive consumption of certain foods [2]. Nonetheless, information is lacking over whether this situation is in accordance with an isolated maladjusted food behaviour or conversely, whether this is due to food components with addictive capacity [3].

Currently, only a tool that enables evaluating FA called Yale Food Addiction Scale (YFAS), is available in the scientific literature [4]. This attempts to operationalize the concept of food addiction by translating diagnostic criteria for substance-related disorders highlighted in the Diagnostic and Statistical Manual of Mental Disorders (DSM) for its application to food behaviour [5].

Against this backdrop, the tool has several versions that have been validated for the adult [4, 6–8], population [9] and paediatric populations, respectively [10]. To date there have been translations and adaptations into Spanish for the adult version [11] and recently the paediatric version called Spanish Yale Food Addiction Scale for Children (S-YFAS-C) was transculturally validated into Spanish [12].

After a process of translation, adaptation and transcultural validation, the S-YFAS-C scale contains 25 questions that enable validating the degree of FA, but also quantifying the symptoms that go undiagnosed in this target population [12]. This work aims to evaluate the tool proposed in a non-clinical pilot sample with the purpose of continuing the process of validation of the scale and according to the original YFAS model [4]. Therefore, considering that the child and juvenile population are more vulnerable to unhealthy food behaviours, in general, and pathological relationships with food specifically, a preliminary evaluation of the degree of FA in this paediatric population is necessary.

## Material and methods

A transversal observational study was performed on a convenience sample comprised of boys and girls aged 9 to 12 attending a private school from the province of Santa Cruz de Tenerife. The study includes students studying 4th to 6th year primary school with the corresponding authorization from the family or legal guardians to take part.

For its implementation, self-administered (ad hoc) data collection was initially established for each student body with demographic data and outcome measures to use (FA evaluation questionnaire, child feeding attitudes questionnaire and visual scale to evaluate body image). An anthropometric evaluation of the student body was subsequently performed by health professionals. Finally, the questionnaire opinion is evaluated in a group of 15 boys and girls accompanied by investigators by means of debriefing methodology [13]. Therefore, words, terms or concepts are identified that respondents do not understand, do not understand systematically or do not interpret what investigators intend [14].

### Result outcomes

- Questionnaire for evaluation of FA in the child population (S-YFAS-C) [12]. The questionnaire is comprised of 25 items and examines food behaviour in regard to the last 12 months based on seven criteria for diagnosis of substance dependence according to the DSM-IV-TR [15]. The scores evaluate FA in two different ways. First, counting symptoms, which offers a score version that reflects the number of dependence symptoms based on the seven criteria outlined without considering the clinical importance on the score (a threshold of three or more symptoms was used to indicate a possible FA disorder). Second, the diagnosis of addiction, which evaluates whether or not FA can be diagnosed. This is confirmed when three or more symptoms and anxiety or significant clinical worsening are present.
- Children Eating Attitudes Test (ChEAT) [16]. This is a tool that enables estimating the prevalence of the risk of presenting a Food Disorder (FD), which facilitates identifying problems aimed at a continuous concern for food, abnormal food models and attitudes at these ages. The questionnaire score range is 0 to 78 and a threshold equal or greater than 20 points is set to consider that a boy or girl presents a risk of developing a FD. This test has been used because it is one of the most widely used in the literature, and has been validated in the study subjects analyzed, presenting a high reliability coefficient.
- Contour Drawing Rating Scale (CDRS) [17]. This is a visual scale that evaluates body image comprised of nine contours of male and female figures, respectively. The contours increase in size with the score and the degree of satisfaction or discrepancy index is obtained by means of the difference between desired and perceived body image. A difference in two points was deemed an abnormality in the image. It is one of the most widely used scales to analyze body perception, with a test–retest reliability of r: 0.78 (*p* < 0.0005), and is used in several studies in both adolescent and adult populations.
- Anthropometric evaluation. Data on weight, height, Body Mass Index (BMI), percentile, basal metabolism, standard deviation and levels of adipose, muscular and aqueous tissue were obtained by means of an approved bioimpedance scale (Tanita BC-418 model).
- Debriefing questionnaire. This is one of the most commonly used techniques to definitively draw up an outcome questionnaire. Its aim is to: (a) identify misunderstood or poorly interpreted words, terms or concepts; (b) identify the issues that cannot be precisely answered and/or where there are queries; (c) obtain suggestions for review of drawing up questions and questionnaire structure.

### Data analysis

The continuous variables analyzed were summarized by means of mean and standard deviation (SD), or as median and 25th and 75th (P25 and P75) percentile, according to their normality; qualitative variables were analyzed as count and percentage. For the bivariate association between continuous variables the student-*t* or Mann–Whitney U test was used according to their normality; and for qualitative variables the Pearson Chi-squared or Fisher exact test was used. Fisher exact test was used when the expected cell size < 5.

The association between Z-score deviation, the S-YFAS-C scale symptoms count and the ChEAT scale score was analyzed by means of Pearson correlations. Analyses were performed with the programme IBM SPSS (Statistical Package for the Social Sciences) version 22.0 (IBM Corp. Armonk, NY. USA). A *P*-value ≤ 0.05 was considered statistically significant. When *P*-values were between 0.05 and 0.1 a trend towards statistical significance was considered.

## Results

Table 1 shows the basal characteristics of the total of 45 boys and girls included in the study. The minimum and maximum values obtained were for age in years (9.48–12.33), weight in kilograms (26.6–64.5), height in centimetres (131–163), BMI (14.2–27.9) and BMI Z-score (−1.36–2.66). Table 1 reveals statistically significant differences by sex in basal metabolism. This is greater in boys (*P* = 0.027) and bone mass is significantly higher in girls (*P* = 0.020). The percentage of fat mass appears higher with a significant tendency in girls (*P* = 0.050). The percentage of body water is higher in boys (*P* = 0.053).

**Table 1:** Basal characteristics of the sample

	Girl (n = 21)	Boy (n = 24)	Total (n = 45)	*P*-value
	Mean (SD)	Mean (SD)	Mean (SD)	
Age	10.66 (0.76)	10.88 (0.81)	10.78 (0.79)	0.359
Weight (kg)	39.22 (9.84)	37.26 (7.99)	38.18 (8.85)	0.466
Height (cm)	145.62 (8.26)	143.83 (7.55)	144.67 (7.85)	0.453
Body Mass Index (BMI)	18:28 (03.12)	17.88 (2.74)	18:07 (2.90)	0.648
Z-score BMI	−0.07 (0.83)	−0.06 (0.89)	−0.07 (0.85)	0.199

	n (%)	n (%)	n (%)	
*Classification according to BMI z-Score*				0.199
Malnutrition (Z-score ≤ −1)	0 (0)	2 (8.3)	2 (4.4)	
Normality (Z-score ≥ −0.99 and SD < 1)	19 (90.5)	18 (75.0)	37 (82.2)	
Overweight (Z-score ≥ 1 and SD < 2)	1 (4.8)	4 (16.7)	5 (11.1)	
Obesity (Z-score ≥ 2)	1 (4.8)	0 (0)	1 (2.2)	

	Mean (SD)	Mean (SD)	Mean (SD)	
Fat mass (kg)	9.86 (4.74)	8.09 (3.50)	8.91 (4.18)	0.158
Fat mass (%)	24.18 (5.14)	21.02 (5.33)	22.49 (5.42)	**0.050**
Fat free mass (kg)	29.38 (5.67)	29.18 (5.18)	29.27 (5.35)	0.904
Total body water (kg)	21.51 (4.15)	21.37 (3.80)	21.43 (3.92)	0.905
Total body water (%)	55.55 (3.78)	57.84 (3.91)	56.77 (3.98)	**0.053**
Basal metabolism (kcal)	1224.10 (151.77)	1321.88 (134.31)	1276.24 (149.45)	**0.027**
Bone mass (kg)	1.36 (0.34)	1.12 (0.33)	1.23 (0.35)	**0.020**
Muscular mass (kg)	28.01 (5.36)	28.06 (4.88)	28.04 (5.05)	0.977

*BMI* Body Mass Index, *SD* standard deviation, *kg* kilograms, *cm* centimetres, *Kcal* kilocalories.

*P*-values in bold are significant values (*P* ≤ 0.05) or values with a tendency to significance (0.05 < *P* ≤ 0.1)

The results obtained by means of the outcome instruments are covered in Table 2. The average number of FA symptoms measured with the S-YFAS-C scale is 1.67 ± 1.45 (range 0–7). A total of 20% of boys and girls present three or more symptoms for FA. The clinically significant diagnosis for worsening or anxiety was not reflected among the student body. No statistically significant differences were found between boys and girls in the scores obtained according to the seven criteria reported in the S-YFAS-C scale (Table 2).

**Table 2:** Distribution of the results obtained by means of applied measurement instruments

	**Girl** **(n = 21)**	**Boy** **(n = 24)**	**Total** **(n = 45)**	***P*-value**
	Mean (SD)	Mean (SD)	Mean (SD)	
*S-YFAS-C*				
Quantification of symptoms	1.62 (1.77)	1.71 (1.12)	1.67 (1.45)	0.839

	n (%)	n (%)	n (%)	
Food addiction^*^				
Yes	4 (19)	5 (20.8)	9 (20)	< 0.999
No	17 (81)	19 (79.2)	36 (80)	

	n (%)	n (%)	n (%)
*S-YFAS-C diagnostic criteria*			
1. The substance ingested in a large amount and for longer than expected	1 (4.8)	2 (8.3)	3 (6.7)
2. Chronic desire or repeated attempts to stop consuming it	5 (23.8)	6 (25)	11 (24.4)
3. Time to obtain and use of the substance or recovery of its effects	3 (14.3)	3 (12.5)	6 (13.3)
4. Social, occupational or recreational aspects that are abandoned or reduced due to the substance	12 (51.1)	16 (66.7)	28 (62.2)
5. Substance use despite knowledge of the adverse consequences (n = 42)	1 (5)	1 (4.5)	2 (4.8)
6. Tolerance (n = 43)	4 (20)	4 (17.4)	8 (18.6)
7. Characteristic abstinence symptoms. Substance consumption to relieve abstinence	8 (38.1)	9 (37.5)	17 (37.8)

	Mean (SD)	Mean (SD)	Mean (SD)	
*ChEAT*				
*Score*	11.76 (10.69)	13.83 (8.57)	12.87 (9.56)	0.475

	n (%)	n (%)	n (%)	
Existence of FD^µ^				
Yes	2 (9.5)	7 (29.2)	9 (20)	0.143
No	19 (90.5)	17 (70.8)	36 (80)	

	Median (P25;P75)			
*CDRS*				
Perceived body image	4 (3; 5)	5 (4; 6)	4 (3.5; 5)	0.100
Desired body image	3 (2.5; 4)	5 (4; 5)	4 (3; 5)	**0.001**

	n (%)	n (%)	n (%)	
Distortion of body image^†^				
Yes	5 (23.8)	4 (16.7)	9 (20)	0.713
No	16 (76.2)	20 (83.3)	36 (80)	

	Mean (SD)	Mean (SD)	Mean (SD)	
Difference between: perceived and desired body image	−1 (1.18)	−0.42 (1.10)	−0.69 (1.16)	**0.094**

*Food addiction: existence of three or more symptoms on the S-YFAS-C scale

µ Existence of food disorder: score ≥ 20 on the ChEAT scale

^†^Distortion of body image: difference ≥ 2 between perceived and desired image

*SD* standard deviation,* FD* food disorder, *S-YFAS-C* Spanish Yale Food Addiction Scale for Children, *ChEAT* Children Eating Attitudes Test, *CDRS* Contour Drawing Rating Scale

*P*-values in bold are significant values (*P* ≤ 0.05) or values with a tendency to statistical significance (0.05 < *P* ≤ 0.1)

A total of 20% of the student body presented a risk of developing a FD measured by means of the ChEAT scale, with an average score of 12.87 ± 9.56 (Table 2). Distortion of body image (measured with the CDRS instrument), was observed in 20% of the sample (the average difference between perceived and desired body image was -0.69 ± 1.16 points).

In the sex comparison of the different instruments, statistically significant differences were only observed between desired body image between boys and girls (*P* = 0.001). Girls desire a thinner image than boys. For the remaining measures no statistically significant differences were detected (Table 2).

Table 3 shows the relationship between distortion of body image and quantification of symptoms for FA and risk scores for FD. Although statistically significant differences were not detected, the student body that suffers distortion of body image shows a higher number of symptoms on the S-YFAS-C scale (2.33 ± 1.94) and a higher risk score for ChEAT (16.11 ± 15.10) compared to the student body that does not show distortion of body image.

**Table 3:** Relationship between distortion of body image and number of symptoms for FA and risk scores for FD

	Distortion of body image		
	Yes (n = 9)	No (n = 36)	*P*-value
	Mean (SD)	Mean (SD)	
S-YFAS-C number of symptoms	2.33 (1.94)	1.50 (1.28)	0.123
Risk score for ChEAT	16.11 (15.10)	12.06 (7.71)	0.260

*SD* standard deviation, *S-YFAS-C* Spanish Yale Food Addiction Scale for Children, *ChEAT* Children Eating Attitudes Test

No correlation was detected between the Z-score deviation according to BMI and the ChEAT tool score with the number of symptoms from the S-YFAS-C scale (r = 0.059; *P* = 0.7 and r = 0.097; *P* = 0.528, respectively). Meanwhile, there was a positive correlation between the symptoms count on the S-YFAS-C scale and the ChEAT scale score (r = 0.595; *P* < 0.001).

As for the comparison of the ChEAT scale score according to FA (three or more symptoms on the S-YFAS-C scale), statistically significant differences were detected (*P* = 0.035) between those with less than three symptoms (10.31 ± 5.38) and those presenting three symptoms or more (23.11 ± 15.13).

In regard to the 15 interviews of the student body by means of debriefing, Table 4 shows the development times for the S-YFAS-C scale, as well as scores for the degree of compliance, interest and simplicity of language. The average time spent was 4.67 min. Questions 3, 7, 8, 9 and 15 were reflected by participants as of interest, whilst questions 1, 12, 17 and 18 were suggested with a query or problems understanding.

**Table 4:** Representation of the student-interviewer intervention by means of “debriefing” methodology

	Girl (n = 8)	Boy (n = 7)	Total (n = 15)
	Mean (SD)	Mean (SD)	Mean (SD)
1. How long did you take to fill this in?			
	5.0 (1.41)	4.29 (1.70)	4.67 (1.54)

	n (%)	n (%)	n (%)
2. Was it too long to fill in?			
No	6 (75)	6 (85.7)	12 (80)
Yes	1 (12.5)	0 (0)	1 (6.7)
More or less	1 (12.5)	1 (14.3)	2 (13.3)
3. Do you think all the questions are of interest?			
No	0 (0)	0 (0)	0 (0)
Yes	5 (62.5)	5 (71.4)	10 (66.7)
More or less	3 (37.5)	2 (28.69)	5 (33.3)
4. Are the questions drawn up in simple language or is there one that sounds like forced use of language?			
No	7 (87.5)	4 (57.1)	11 (73.3)
Yes	1 (12.5)	0 (0)	1 (6.7)
More or less	0 (0)	3 (42.9)	3 (20)

*SD* standard deviation

## Discussion

The evaluation made to evaluate the degree of FA by means of the S-YFAS-C scale has enabled piloting its first application in a non-clinical sample from the Spanish paediatric population. This is the first study to apply this tool in a healthy student body sample and enables studying its validation after having been culturally translated and adapted.

The original YFAS-C scale was validated by its authors [10] in a sample of 75 boys and girls. A mean count of the seven symptoms of 2 ± 1.81 was obtained. This version in Spanish was drawn up on a sample of 45 students and a very similar average was obtained when the symptoms were counted (1.67 ± 1.45). This fact suggests that the Spanish version can be applied in a valid manner in paediatric populations. In fact, the counting of symptoms scoring option is the most sensitive measure to evaluate subclinical food behaviours. As reflected by its original authors [10], the youngest samples may be less likely to comply with the full diagnostic criteria for FD.

To be able to validate the instrument other measures have been applied such as the ChEAT tool, which enables identifying the risk of suffering from a FD [16], or scales such as the CDR that evaluate distortion of body image [17]. Most validations that have been performed on this original scale into different languages apply these tools to obtain correlations between symptoms related to existence of FD. They also use variables that identify significant differences between ages and sexes, aside from anthropometric evaluation data [18].

In principle, in the sample studied no statistically significant differences were identified between the sociodemographic variables. However, there were differences in the data obtained for body composition. It is worth noting that 82.2% of the sample had normal weight according to the BMI. According to the data obtained in the literature, the prevalence of FA is more frequent among boys and girls with excess weight [19]. This may justify that the clinically significant diagnosis of worsening or anxiety will not be reflected in any subject. However, 20% will present three or more symptoms of FA.

The sample studied presents a 20% risk of developing a FD, as observed in Table 2. This data is similar to studies performed in the same community by other authors where a figure of 18.2% is obtained in terms of risk of presenting this pathology [20]. In this context, distortion of body image is also observed in 20% of the subjects studied. Moreover, statistically significant differences were observed between desired body image in boys and girls (*P* = 0.001) as also reflected by the original authors of the YFAS [10]. In general, it was observed that the student body desires a thinner image than what they show. There is a difference in average between the perceived and desired body image of −0.69 ± 1.16 points. This is greater in the case of girls (61.9% of girls seek a thinner figure compared to 41.7% of boys).

However, as outlined previously, virtually the entire sample showed a healthy weight and this facilitates being unable to set out differences with the outcome measures applied and the BMI. Therefore, no differences were observed between the Z-score deviation according to BMI and the symptoms count or ChEAT tool score.

Nonetheless, there was a positive correlation between the S-YFAS-C scale score and the ChEAT scale score. In fact, the student body that presented three or more symptoms for food addiction, obtained a higher score on the ChEAT instrument than the student body presenting under three symptoms. These statistically significant differences suggest that the S-YFAS-C instrument is not only detecting a possible food addiction as such, but also identifying the risk of suffering from a FD. In short, both tools are valid and can be analyzed to guide and identify anomalous food behaviours between young and juvenile populations.

In regard to understanding the questionnaire that was validated, it was observed that the difficulty to fill this in, development and understand it is simple, as can be observed in Table 4. Only questions 1, 12, 17 and 18 suggest some queries or problems understanding and this has facilitated partially amending to improve the language even further. The possibility of undertaking the interview jointly with the 15 boys and girls enabled identifying these issues, thereby facilitating final preparation of the questionnaire.

Finally, this work has limitations given the inherent nature of its design. In summary, this is an initial pilot with a very reduced convenience sample that does not enable obtaining more relationships than those shown. Moreover, these are healthy subjects that comprise a school with a high sociocultural level. This may have had an impact on the data obtained and it would have been appropriate to analyze the family’s sociocultural level, as well as the availability of financial resources. In the other hand, it would also have been interesting to know the parents' perception of their children's possible addiction to food. This fact should be considered in future studies to assess it as a possible new validation criterion.

In short, despite being an initial pilot and its limitations, the S-YFAS-C scale applied in its setting was initially evaluated. In future studies it is suggested applying the tool in clinical patients diagnosed with excess weight; but also in patients diagnosed with FD as suggested by the study’s original authors [10].

## Conclusions

The previously validated S-YFAS-C scale and now implemented in a paediatric pilot sample enables satisfactorily evaluating food addiction in boys and girls. This tool will enable diagnosing food addiction in Spanish-speaking boys and girls. The number of symptoms score is the most sensitive measure shown to evaluate subclinical food behaviours related to this addiction.

## Data Availability

The datasets generated during and/or analysed during the current study are not publicly available due to ethical restrictions in order to protect the confidentiality of the participants, but are available from the corresponding author on reasonable request.

## Abbreviations

BMI: Body Mass Index
ChEAT: Children Eating Attitudes Test
CDRS: Contour Drawing Rating Scale
DSM: Diagnostic and Statistical Manual of Mental Disorders
FA: Food Addiction
FD: Food Disorder
S-YFAS-C: Spanish Yale Food Addiction Scale for Children
SD: Standard Deviation
YFAS: Yale Food Addiction Scale
